# Global Extension and Predominance of Human Metapneumovirus A2 Genotype with Partial G Gene Duplication

**DOI:** 10.3390/v14051058

**Published:** 2022-05-16

**Authors:** Juan Carlos Muñoz-Escalante, Gabriel Mata-Moreno, Gerardo Rivera-Alfaro, Daniel E. Noyola

**Affiliations:** 1 Microbiology Department, Facultad de Medicina, Universidad Autónoma de San Luis Potosí, San Luis Potosí 78210, Mexico; carlos.escalante@uaslp.mx (J.C.M.-E.); a238871@alumnos.uaslp.mx (G.R.-A.); 2 Section of Molecular and Translational Medicine, Centro de Investigación de Ciencias de la Salud y Biomedicina, Universidad Autónoma de San Luis Potosí, San Luis Potosí 78210, Mexico; a188504@alumnos.uaslp.mx

**Keywords:** human metapneumovirus, epidemiology, acute respiratory infections, genotype, molecular epidemiology, pneumovirus

## Abstract

Human metapneumovirus (HMPV) is an important respiratory pathogen and is divided in two main groups (A and B). HMPV strains with partial duplications (111-nt and 180-nt duplication) of the G gene have been reported in recent years. Since the initial reports, viruses with these characteristics have been reported in several countries. We analyzed all complete HMPV G gene ectodomain sequences available at GenBank to determine if viruses with 111-nt or 180-nt duplication have become the leading HMPV strains worldwide, and to describe their temporal and geographic distribution. We identified 1462 sequences that fulfilled study criteria (764 HMPV A and 698 HMPV B) reported from 37 countries. The most frequent HMPV A genotype was A2b2 (*n* = 366), and the most frequent B genotype was B2 (*n* = 374). A total of 84 sequences contained the 111-nt duplication, and 90 sequences contained the 180-nt duplication. Since 2016, viruses with a partial duplication comprise the most frequent HMPV A sequences globally and have displaced other HMPV A viruses in Asia, Europe, and South America; no sequences of viruses with partial duplication have been reported in North America or Africa so far. Continued surveillance of HMPV is required to identify the emergence and spread of epidemiologically relevant variants.

## 1. Introduction

Human metapneumovirus (HMPV) is an important causative agent of upper and lower respiratory infections in children and adults. This virus was first reported in 2001, although viral circulation can be traced back to the 1950s [1]. About half of children are infected by HMPV by two years of age, and by the age of five most children have already been infected by this virus. Infections caused by this pathogen are usually mild to moderate in healthy individuals; however, numerous reports have shown severe and even fatal pneumonia can occur, predominantly in elderly individuals and immunocompromised patients [2].

HMPV is an enveloped, negative-sense, single-stranded RNA virus that is classified, together with respiratory syncytial virus (RSV), in the Pneumoviridae family. The viral genome contains eight genes (N, P, M, F, M2, SH, G, and L), which encode a total of nine proteins, including three surface glycoproteins: F (fusion), SH (small hydrophobic), and G (attachment) [2].

HMPV strains are divided in four major genotypes: A1, A2, B1, and B2. The G protein gene is the most variable of all viral genes and mutations occur predominantly in the region encoding for the extracellular domain of the protein [3]. Diversity of HMPV has led to further divisions within genotype A2. In 2006, Huck et al. first described two distinct clades of A2 viruses, termed A2a and A2b [4]. Since then, A2b viruses have been shown to cluster in two main clades which have been referred to by different names: A2b1, the clade which includes older sequences and continues to be referred to as A2b by many authors and more recently as A2.2.1; and A2b2, the clade which encompasses most recently isolated viruses, including viruses with partial duplications of the G gene, which is referred to as A2c by many authors, but also referred to as Novel A2 sublineage/Unique A2 sublineage/Unique A2b sublineage and more recently A2.2.2 [3,5,6,7,8,9,10,11,12,13,14,15]. Of interest, in 2017 HMPV strains with partial duplication of the G gene were described [7,16]. These include two distinct groups of viruses: those with a 111 nt duplication and those with a 180 nt duplication [7,16,17]. Since then, viruses carrying these partial duplications of the G gene have been identified in China, Croatia, and other countries [8,9]. Of note, some genotypes of RSV, a virus closely related to HMPV, have also developed partial duplications in the G gene, including RSV B viruses (described initially as BA genotype) and RSV A viruses (described as ON1 genotype) [18,19]. Since their identification, BA and ON1 viruses have become the predominant RSV strains, making previous genotypes almost undetectable around the world in recent years [20]. Recent research suggests the existence of an evolutive advantage linked to partial duplications in the G gene; however, the mechanisms leading to the predominance of these viruses are still under study. In addition, the development of partial duplication in both RSV and HMPV suggests that this may be a general evolutive mechanism in this family of viruses.

In light of this, in the present study we analyzed all HMPV sequences (whole genomes, partial genomes, complete coding sequences, and partial coding sequences) that included the G gene ectodomain region available at GenBank up to December 2021 to assess if viruses with partial duplication of this gene have become the leading HMPV viruses worldwide. In addition, we aimed to provide updated information regarding the phylogenetic relationships between HMPV strains reported globally.

## 2. Materials and Methods

Sequence acquisition: All HMPV sequences available on GenBank up to 2 December 2021, were downloaded. Obtained sequences included whole genomes, partial genomes, complete gene coding sequences and partial gene coding sequences. Synthetic sequences, chimeric sequences, and those corresponding to organisms or viruses different from HMPV were excluded. For each sequence, temporal and geographic data (date and country of sample collection) were registered.

Genotype Reference Sequence Selection: We performed an extensive literature review of articles describing HMPV molecular epidemiology and registered 327 HMPV sequences used as genotype references in 22 different articles; 19 of these articles focused on genotype assignment using the HMPV G gene. Subsequently, we selected sequences that have been used as genotype reference by several authors or sequences of newly identified genotypes for viruses with partial G gene duplications [16,21,22,23,24,25,26,27,28,29,30,31,32,33]. As our aim focused on genotype assignment using the HMPV G gene ectodomain, sequences with at least the complete ectodomain were selected as references. A total of 44 HMPV A (10 A1, 8 A2a, 12 A2b1, 2 A2b2, 3 A2b2-111, and 4 A2b2-180) and 49 HMPV B (25 B1 and 24 B2) sequences comprised the genotype reference dataset (Supplementary Table S1).

HMPV G ectodomain sequence curation. A total of 10,088 HMPV sequences underwent local BLAST against an HMPV G gene Genotype Reference library using Blast2Go v6.0 (BioBam Bioinformatics, Valencia, Spain) to identify and extract sequences spanning the HMPV G gene; a total of 2538 sequences that contained the HMPV G gene were identified. Once selected, these sequences were aligned using the MUSCLE algorithm and manually cropped to the HMPV G gene ectodomain length. Sequences shorter than the ectodomain length (500 nt) (953 sequences), sequences with insertions or deletions different from the 111 and 180 nucleotide duplications (6 sequences), and sequences containing undetermined or degenerate nucleotides (60 sequences) were removed. In total, 1519 HMPV G gene ectodomain sequences (765 HMPV A and 754 HMPV B) fulfilled the criteria required for subsequent genotype assignment analysis.

Genotype assignment: Genotype assignment was based on the analysis of 1301 unique sequences (686 HMPV A and 615 HMPV B) derived from the 1519 sequences previously described and was carried out by clustering with 44 HMPV A and 49 HMPV B genotype reference sequences in a cladogram generated by a Maximum Likelihood analysis under the GTR nucleotide substitution model with invariant site rates, gamma distribution and 1000 iterative resampling using MEGA software v10.2.6 (MEGA Software Development Team). The number of sequences (total and for each genotype) reported for each year were recorded for descriptive analysis. Global results, as well as sequences reported in each continent (Africa, Asia, Europe, North America, Oceania, and South America) were analyzed.

Recombination event detection: In order to maximize the accuracy of the phylogenetic analysis, a recombination detection analysis was performed using RDP5 v5.5. Possible recombination events were evaluated using RDP, GENECONV, Chimaera, MaxChi, SiScan, 3Seq, and LARD algorithms; additionally, the online tool GARD (https://www.datamonkey.org/gard accessed on 4 March 2022) was used to evaluate possible recombination events. True recombination events were defined as those detected for at least four different algorithms using RDP5 and additionally detected by GARD. Sequences with evidence of recombination events were removed from the dataset.

## 3. Results

**Figure 1:**
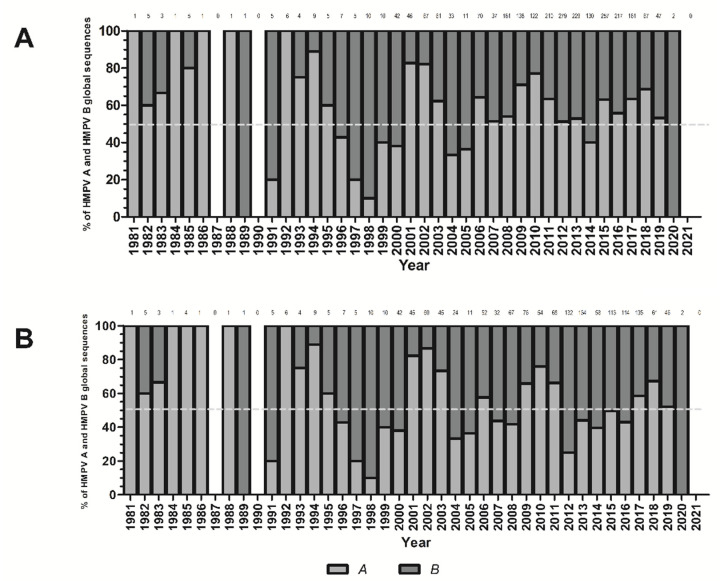
Proportion of human metapneumovirus (HMPV) A and HMPV B sequences available globally for each year between 1981 and 2021, including all 2538 HMPV G gene sequences identified in the GenBank database fulfilling the study acquisition inclusion criteria (**A**) and the 1519 HMPV G gene sequences for which genotype assignment could be carried out (**B**). The number of sequences identified during a given year is shown on top of each bar; no sequences for 1987, 1990, and 2021 were identified.

Overall, 2538 HMPV G gene sequences were identified. 1453 were assigned as HMPV A and 1085 as HMPV B based on the local BLAST analysis performed using Blast2Go v6.0 against HMPV A and HMPV B reference sequences libraries. However, 1019 (40.15%) of these sequences were excluded from genotype assignment because they did not include the complete ectodomain, or due to the presence of ambiguities or insertions/deletions other than the 111 and 180 nucleotide duplication. Genotype assignment was carried out for the remaining 1519 (59.85%) sequences (765 HMPV A and 754 HMPV B). The temporal distribution of HMPV A and HMPV B G gene sequences was comparable when all available sequences (*n* = 2538) were compared with those in which genotype assignment analysis could be carried out (*n* = 1519) as shown in Figure 1.

**Figure 2:**
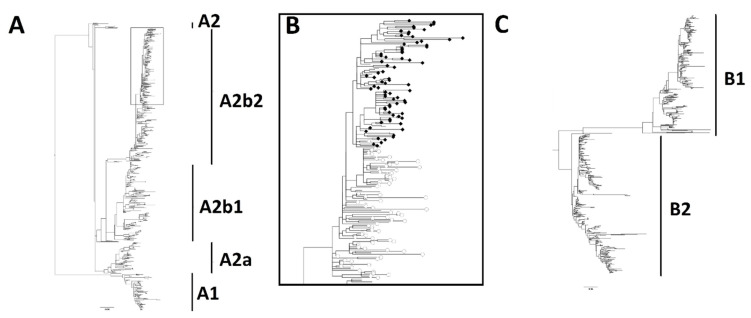
Phylogenetic trees of 765 unique human metapneumovirus (HMPV) A (**A**,**B**) and 751 unique HMPV B (**C**) complete G gene ectodomain sequences generated by a Maximum Likelihood analysis with the use of 44 and 49 reference sequences, respectively. The box in panel (**A**) is enlarged in panel (**B**), where A2b2 sequences with 111-nt (closed diamonds) and 180 nt (open circles) partial duplications can be identified.

Genotype assignment was performed based on cladistic analysis of those samples from which the complete ectodomain sequence was available (Figure 2). Genotype identification was possible for 1516 out of 1519 sequences which were subjected to analysis (765 HMPV A and 751 HMPV B); three samples did not cluster within any of the genotypes. Recombination analysis suggested the presence of true recombination events in these sequences; we could not determine if these represent real recombination events, the presence of coinfections, or artifacts generated during sequence assembly. These sequences were excluded from further analysis. Complete sample collection data (year and country of isolation) was available for 1462 sequences: 764 HMPV A (99.87%) and 698 HMPV B (92.94%) which comprised the final study dataset.

Sequences included in the study were identified in 37 different countries distributed on six continents/subcontinents (Africa, Asia, Europe, North America, Oceania, and South America) (Supplementary Table S2). The first available HMPV sequence was collected in the Netherlands in 1981 and assigned as HMPV A genotype A2 (GenBank accession number AY296022) [34]. Spain accounted for the largest set of sequences (*n* = 162; 49 HMPV A and 113 HMPV B) followed by Japan (*n* = 146; 86 HMPV A and 60 HMPV B), Canada (*n* = 145; 87 HMPV A and 58 HMPV B), China (*n* = 131; 91 HMPV A and 40 HMPV B), the United States (*n* = 107; 64 HMPV A and 43 HMPV B), and Kenya (*n* = 99, 52 HMPV A and 47 HMPV B). Countries were grouped by continent and Asia accounted for the largest set of sequences (*n* = 500; 284 HMPV A and 216 HMPV B) followed by Europe (*n* = 294; 120 HMPV A and 174 HMPV B), Africa (*n* = 260; 105 HMPV A and 155 HMPV B), North America (*n* = 256; 152 HMPV A and 104 HMPV B), South America (*n* = 103; 76 HMPV A and 27 HMPV B) and Oceania that accounted for the smallest set (*n* = 49; 27 HMPV A and 22 HMPV B).

**Figure 3:**
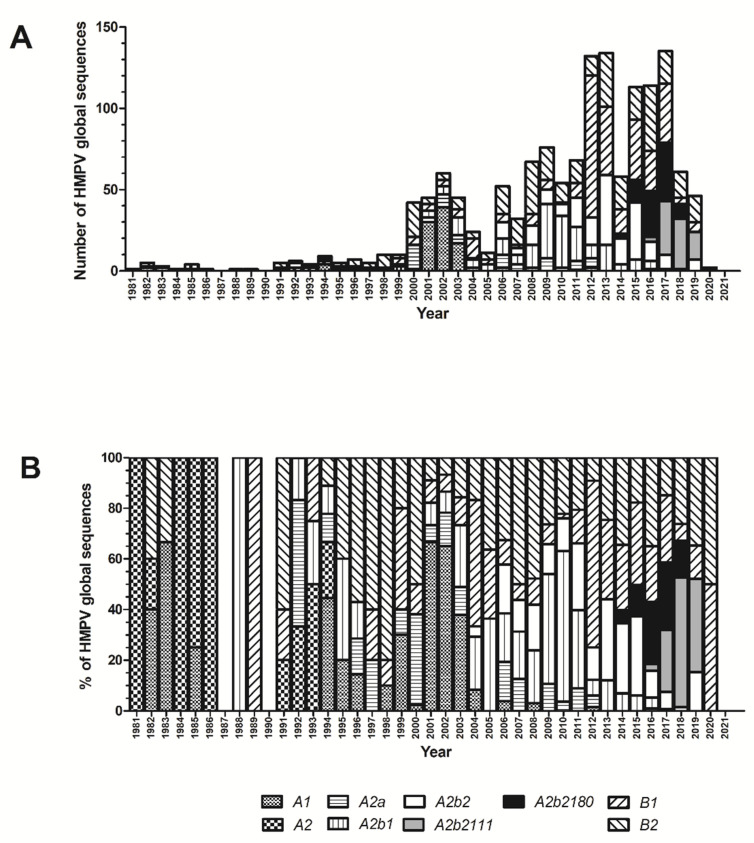
Number (**A**) and proportion (**B**) of global human metapneumovirus (HMPV) sequences assigned to each genotype during each year based on complete G gene ectodomain analysis for sequences available at GenBank up to December 2021. Sequences corresponding to the A2b2 genotype that contain the 111- or the 180-nucleotide duplication are represented in different solid colors.

At least one sequence was available for all years, except 1987, 1990, and 2021, when no sequences were identified (Figure 3). The yearly number of sequences was small before the year 2000, with a substantial increase in available sequences during the last two decades.

**Figure 4:**
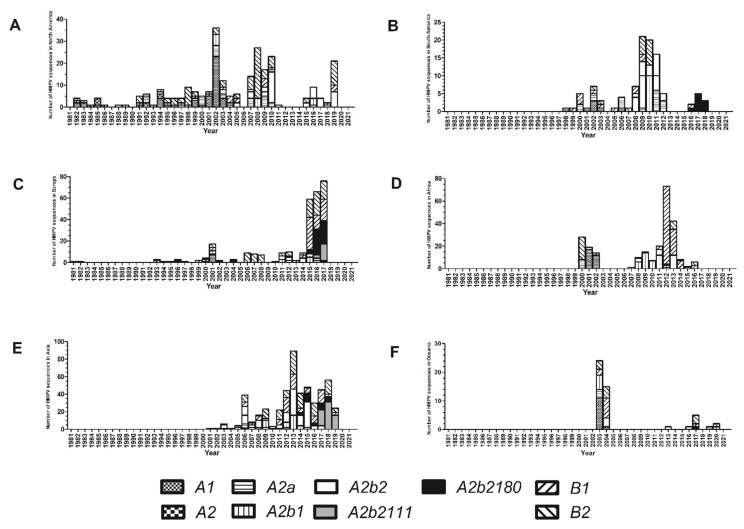
Number of human metapneumovirus (HMPV) sequences assigned to each genotype during each year in North America (**A**), South America (**B**), Europe (**C**), Africa (**D**), Asia (**E**), and Oceania (**F**) based on complete G gene ectodomain analysis for sequences available at GenBank up to December 2021.

**Figure 5:**
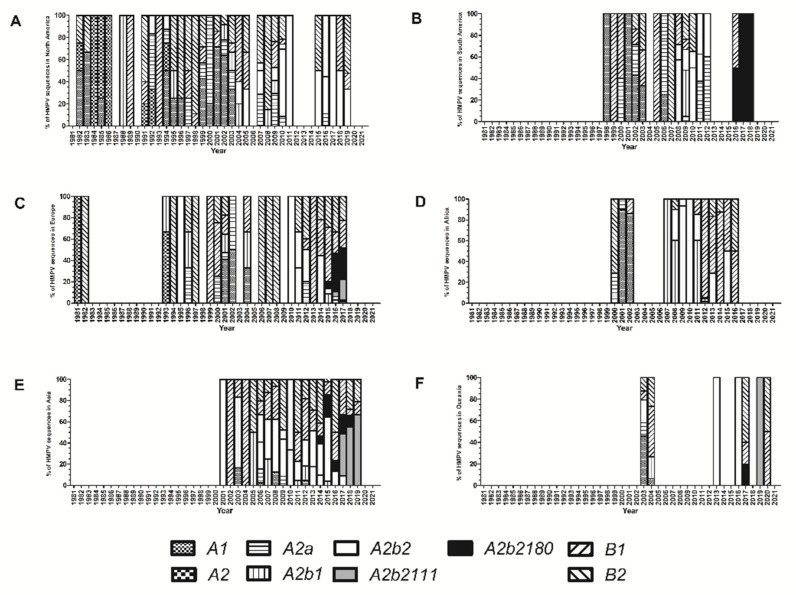
Proportion of human metapneumovirus (HMPV) sequences assigned to each genotype during each year in North America (**A**), South America (**B**), Europe (**C**), Africa (**D**), Asia (**E**), and Oceania (**F**) based on complete G gene ectodomain analysis for sequences available at GenBank up to December 2021.

Between 1981 and 1986, genotypes A1 and A2 predominated; as previously noted, the number of sequences in those years was small, and this should be taken into account when interpreting these results. After 1992, co-circulation of HMPV A and B was observed during all years (except in 2020; of note, at the time of this analysis, only two sequences from this year have been reported), and multiple A or B genotypes have been identified each year. Co-circulation of multiple genotypes during a given year occurs in all continents (Figure 4 and Figure 5). However, the predominant genotype during a given year varies in different continents. Notwithstanding this observation, comparisons between all continents are not possible because HMPV genotype information is not available for all years in each continent, and in some of them (such as Oceania) data is available only for a few years.

Globally, the most frequent HMPV A genotype was A2b2 (*n* = 366, 47.84%), followed by A2b1 (*n* = 201, 26.27%), A1 (*n* = 109, 14.27%), and A2a (*n* = 74, 9.67%). Additionally, 14 sequences clustered inside the A2 clade but did not correspond to any of the three genotypes (A2a, A2b1, or A2b2); these HMPV viruses circulated between 1981 and 1994 and were assigned as A2 genotype, comprising 1.83% of HMPV A sequences. For HMPV B, the most frequent genotype was B2 (*n* = 374, 53.58%), followed by B1 (*n* = 324, 46.42%). Geographic distribution of the genotypes did not show a restricted location pattern since sequences of each of the genotypes were identified in every continent.

The earliest sample of HMPV A2b2 was collected in 2006 in China and, since then, has been identified in all continents. From 2017 onward, all HMPV A sequences included in our analysis belong to this genotype. A total of 84 sequences within this genotype contain the 111-nucleotide partial G gene duplication and 90 sequences contain the 180-nucleotide partial G gene duplication. The first sequences that contain the 180-nucleotide partial duplication date back to 2014; since 2016 viruses with a partial duplication comprise the majority of global HMPV A sequences. However, A2b2 viruses with partial duplication of the G gene have not been reported so far in North America or in Africa, while they appear to have totally displaced other HMPV A viruses in Asia, Europe, and South America.

## 4. Discussion

In the present study, we analyzed all HMPV sequences that included the G gene ectodomain region available at GenBank up to December 2021 to assess if viruses with partial duplication of this gene have become the leading HMPV viruses worldwide.

Several HMPV genotypes usually co-circulate in each epidemic season with one genotype being the most prevalent in every given season. The most frequently observed pattern is the co-circulation of three or more HMPV genotypes with none of them showing a prevalence of more than 50% of the sequences. Before the year 2000, a single genotype predominated in several years (more than 50% of sequences); however, the number of sequences during that period was much smaller than thereafter, and the observed predominance of a single genotype might be conditioned by a small sample size. Previous studies have reported that co-circulation of several HMPV genotypes during a given season is a common occurrence [25,35]. An investigation carried out in Kenya concluded that a genotype predominating in one country in a given year was not necessarily the predominant genotype in other countries [6]. This indicates that a HMPV genotype could become the most prevalent in a specific area, but not necessarily represent the predominant circulating genotype in a broader region. This epidemiological behavior has been reported for other respiratory viruses, such as RSV [20].

Overall, a gradual shift in the dominant HMPV genotypes has been observed over the years. Between 1981 and 1986, only A1, A2, and B2 genotypes were identified. Starting in 1991, other genotypes such as A2a, A2b1, and B1 began to co-circulate. The first available A2b2 genotype sequence can be traced back to 2006. Since then, it has become the dominant HMPV A genotype. Although A1 genotype dominated in several years, overall A1 viruses are detected less frequently than other genotypes in most countries. In Germany, infections caused by A1 genotype were identified between the 2000–2001 and 2002–2003 winter seasons, but not between 2003–2004 and 2009–2010 [36]. A study in Italy identified only one A1 genotype virus among 49 children with HMPV infection, while Kong et al. identified one case among 145 HMPV infections that were genotyped in China [37,38]. Many recent studies have failed to detect HMPV A1 cases indicating that this genotype might have become extinct [5,6,7,8,9,10,11,12,13,14,15,16,17]. The 180-nucleotide duplication in the G gene of HMPV was reported to be present in respiratory samples obtained in 2014 and onward in Japan and Spain [7,16]. Subsequently, the 111-nucleotide duplication in the G gene of HMPV was reported by Saikusa et al. in viruses detected in 2017 in Japan [17]. Of note, our results show that these viruses account for the largest proportion of HMPV A sequences globally from 2016 onward.

Partial duplications of the G gene have been also reported for RSV and this feature has been hypothesized to provide an evolutive advantage [27]. This is also likely to be the case for HMPV, since viruses with a partial duplication currently comprise the majority of HMPV A strains. However, in contrast to RSV A viruses with a partial duplication of the G gene (ON1 viruses), which spread to virtually all continents within three years from their initial identification [39], HMPV viruses with a partial duplication have not been reported in Africa or North America so far. At present, HMPV viruses with either the 111-nt or 180-nt duplication have been reported in Australia, Brazil, China, Croatia, India, Japan, and Spain. Continued molecular surveillance studies are required to assess the global distribution of viruses with these duplications over the following years.

It is worth mentioning that several HMPV molecular epidemiological studies that have been published recently do not allow for the assessment of whether or not HMPV strains with partial duplication of the G gene are circulating in those regions, since phylogenetic analyses target the F gene [30,40,41]. Although there might be molecular markers outside of the G gene associated with the partial duplication of this gene, at present there is no data to support the identification of A2b2 viruses with this characteristic without sequence information for the G gene. As such, it is advisable to include G gene sequencing in future HMPV molecular studies.

Overall, the number of HMPV A and B sequences in which genotype identification was possible were similar, with HMPV A accounting for 50.5% (765/1516) and HMPV B 49.5% (751/1516) of sequences included in the final analysis. HMPV follows a seasonal circulation that peaks in the winter months [26]. HMPV A and B predominance varies depending on the season. Before the year 2012, predominance of HMPV A or B lasting 2 to 4 years was observed: HMPV A predominated between 1992 and 1995; then, the number of HMPV B cases increased, predominating between 1996 and 2000; subsequently, HMPV A predominated again from 2001 to 2003; and, finally, HMPV B surpassed HMPV A in 2004 and 2005. From 2006 onward, the predominance of A or B was less apparent and, overall, both subtypes tend to have a more balanced number of cases. An issue to take into consideration when assessing the proportion of HMPV A and HMPV B detections in the first studies that were carried out after the identification of HMPV is the sensitivity of available molecular assays at that time. The first HMPV strain to be fully characterized (HMPV isolate NL-00-1; GenBank accession number AF371337) corresponds to the A1 genotype [1,34]. As a result, early molecular assays showed a higher sensitivity for detection of HMPV A than HMPV B [42,43]. As such, this may have favored the detection of HMPV A strains compared to HMPV B strains. Nevertheless, because in many studies conserved regions of the genome have been used as detection targets, and thanks to the increasing availability of sequence information, current assays allow for the detection of all HMPV genotypes/lineages.

A limitation of this study is that HMPV genotype could not be assigned to 1021 sequences of the 2538 total because the complete ectodomain region was not available, or because of ambiguous nucleotides or insertion/deletions. However, when the proportion of HMPV A and B sequences throughout the years were compared between all sequences and those sequences in which genotype could be assigned, the observed patterns were similar. Another limitation is that the number of sequences available for each continent is not comparable, and for several continents there are many years for which no sequences were available. Nevertheless, our analysis included all available complete G gene ectodomain sequences available from GenBank.

## 5. Conclusions

In conclusion, A2b2 has become the leading HMPV A genotype over the last two decades, and A2b2 viruses with a partial duplication of the G gene comprise the largest proportion of HMPV A viruses currently circulating in Asia, Europe, and South America. HMPV viruses with partial duplication of the G gene are yet to be reported in Africa and North America. Continued surveillance of HMPV infections is required to identify the emergence and spread of epidemiologically relevant variants.

## Data Availability

This study was based on data available at GenBank (https://www.ncbi.nlm.nih.gov/genbank/ accessed on 2 December 2021).

## Supplementary Materials

The following supporting information can be downloaded at: https://www.mdpi.com/article/10.3390/v14051058/s1, Table S1: List of sequences used as genotype references; Table S2: Number of sequences identified per country (total and according to group and genotype). File S1: HMPV A G gene ectodomain sequence alignment and File S2 HMPV B G gene ectodomain sequence alignment.
