# Supplementary material for: Global Extension and Predominance of Human Metapneumovirus A2 Genotype with Partial G Gene Duplication
Source: Viruses. 2022 May 16;14(5):1058. doi: 10.3390/v14051058 (PMC9146545; doi:10.3390/v14051058)
Supplement: Supplementary file 1 [file viruses-14-01058-s001.zip › viruses-1676363-Supplementary.pdf]

viruses

MDPI

Supplementary Materials

Global Extension and Predominance of Human Metapneumovirus A2 Genotype with Partial G Gene Duplication

Juan Carlos Muñoz-Escalante [1], Gabriel Mata-Moreno [2], Gerardo Rivera-Alfaro [1] and Daniel E. Noyola [1,*]

Table S1. List of sequences used as genotype references

Genotype	Accession Number	Strain	Author
A1	AY296015	FL/4/01	Kenmoe et al. [21]
A1	AY296017	FL/8/01	Zhang et al. [22]
A1	AY485232	hMPV13-2000	Agrawal et al. [23]
A1	AY530092	JPS03-180	Kenmoe et al. 2020 [21]
A1	AY574224	CAN99-81	Zhang et al. 2012 [22]
A1	AY848909	RSA/54/01	Kenmoe et al. 2020 [21]
A1	DQ362950	Arg/2/02	Kenmoe et al. 2020 [21]
A1	EF571502	CHN03-06	Zhang et al. 2012 [22]
A1	JF929839	TN96-12	Zhu et al. 2020 [24]
A1	KU821121	BJ-1610	Perchetti et al. 2021 [25]
A2a	AB503857	Jpn03-1	Al-Turab et al. 2015 [26]
A2a	AY848918	RSA/48/00	Kenmoe et al. 2020 [21]
A2a	DQ362952	Arg/3/00	Kenmoe et al. 2020 [21]
A2a	GQ888741	UY/18/06	Kenmoe et al. 2020 [21]
A2a	JQ309677	SIN06-NTU271	Zhu et al. 2020 [24]
A2a	KC403981	HMPV/AUS/146892777/2003/A	Rahman et al. [27]
A2a	KC403982	HMPV/AUS/143003542/2003/A	Zhu et al. [24]
A2a	KF686742	HMPV/ARG/107/2002/A	Amer [28]
A2b1	AY530091	JPS03-178	Amer [28]
A2b1	AY530093	JPS03-187	Ábrego et al. [29]
A2b1	AY530095	JPS03-240	Ábrego et al. [29]
A2b1	EF571504	CHN05-06	Saikusa et al. [16]
A2b1	EF571506	CHN07-06	Zhang et al. [22]
A2b1	GQ153651	HMPVgz01	Amer [28]
A2b1	JF929848	TN95-354	Kenmoe [21]
A2b1	KC562240	AUS/134249451/2003	Jallow et al. [30]
A2b1	KC731491	HMPV/PUNE/NIV0927648/09	Kenmoe et al. [21]
A2b1	KC731524	PUNE/NIV11442/11	Kenmoe et al. [21]
A2b1	KF178976	H0809-047-A	Saikusa et al. [16]
A2b1	KF178997	C0910-1072	Jallow et al. [30]
A2b1	KU176101	Iran/54/2015	Zhu et al. [24]
A2b1	KU320927	MY/U3700/2014	Zhu et al. [24]
A2b1	KU375595	HR18673-11	Saikusa et al. [16]
A2b1	KU375603	HR1806-12	Saikusa et al. [16]
A2b1	LC192203	Yokohama.JPN/P8386/2016	Zhu et al. [24]
A2b2 111	LC275891	Yokohama.JPN/P8943/2017	Saikusa et al. [31]

A2b2 111	LC466049	Yokohama.JPN/P9576/2018	Saikusa et al. [31]
A2b2 111	LC466054	Yokohama.JPN/P9676/2018	Saikusa et al. [31]
A2b2 180	KX829101	NSVH2016-08-73359	Saikusa et al. [31]
A2b2 180	KX829113	NSVH2016-15-56796	Saikusa et al. [31]
A2b2 180	LC192249	Yokohama.JPN/P7886/2015	Saikusa et al. [31]
A2b2 180	LC466061	Yokohama.JPN/P9577/2018	Saikusa et al. [31]
A2b2	MF045424	MF045424/USA/SC3141	Rahman et al. [27]
A2b2	MH918033	HR577-12	Jallow et al. [30]
B1	AY296034	NL/1/99	Zhang et al. [22]
B1	AY530089	JPS02-76	Kenmoe et al. [21]
B1	AY530094	JPS03-194	Agrawal et al. [23]
B1	AY848859	RSA/23/02	Kenmoe et al. [21]
B1	DQ362958	Arg/1/00	Zhang et al. [22]
B1	EF571511	CHN12-06	Zhang et al. [22]
B1	GQ888739	AR/C+	Agrawal et al. [23]
B1	JF929865	TN89-713	Kenmoe et al. [21]
B1	JX082178	CQ1254	Kenmoe et al. [21]
B1	KC562219	HMPV/USA/C2-175/2005/B	Zhu et al. [24]
B1	KC562230	HMPV/AUS/159148534/2004/B	Amer [28]
B1	KC731513	PUNE/NIV11361/11	Saikusa et al. [16]
B1	KF179002	SYN0405-070	Saikusa et al. [16]
B1	KF179043	C0910-1014-B	Zhu et al. [24]
B1	KF530169	AUS/139983318/2003	Saikusa et al. [16]
B1	KF530179	AUS/133875417/2003	Saikusa et al. [16]
B1	KJ627383	PER/FLA4809/2008	Amer [28]
B1	KU176106	Iran/110/2015	Saikusa et al. [16]
B1	KU320952	MY/U167/2012	Zhu et al. [24]
B1	KU320972	MY/U3417/2014	Zhu et al. [24]
B1	LC192204	Yokohama.JPN/P6865/2013	Zhu et al. [24]
B1	LC192212	Yokohama.JPN/P8398/2016	Zhu et al. [24]
B1	MH918047	HR547-15	Jallow et al. [30]
B1	MT118718	2019_2910	Ji et al. [32]
B1	AY485242	hMPV33-2001	Agrawal et al. [23]
B2	AY296040	NL/1/94	Agrawal et al. [23]
B2	AY296042	NL/1/96	Saikusa et al. [16]
B2	AY296043	NL/6/97	Saikusa et al. [16]
B2	AY297748	CAN98-75	Galiano et al. [33]
B2	AY848863	RSA/3/00	Kenmoe et al. [21]
B2	DQ843658	BJ1816	Jallow et al. [30]
B2	EF535506	TW05-00125	Amer [28]
B2	GQ888743	UY/1/07	Agrawal et al. [23]
B2	JF929874	TN89-356	Saikusa et al. [16]
B2	JF929875	TN91-316	Saikusa et al. [16]
B2	JF929876	TN92-1031	Saikusa et al. [16]
B2	JF929886	TN93-413	Kenmoe et al. [21]
B2	JF929888	TN93-616	Zhu et al. [24]
B2	JF929902	TN85-123	Saikusa et al. [16]
B2	JQ513477	HMPV/Athens.GRC/367.07	Kenmoe et al. [21]
B2	JQ513479	HMPV/Athens.GRC/181.08	Kenmoe et al. [21]

B2	JQ513484	Athens.GRC/1106.08	Saikusa et al. [16]
B2	KC731487	HMPV/PUNE/NIV0926737/09	Kenmoe et al. [21]
B2	KF179030	H0708-195-B	Zhu et al. [24]
B2	KF179041	H0910-175	Jallow et al. [30]
B2	KU320934	MY/U768/2012	Zhu et al. [24]
B2	KU375596	HR18786-11	Saikusa et al. [16]
B2	KU375598	HR22-11	Saikusa et al. [16]
B2	LC192222	Yokohama.JPN/P6922/2013	Zhu et al. [24]

Table S2. Number of sequences identified per country (total and according to group and genotype).

Country	ISO 3166	Continent	Sequences	A	A1	A2	A2a	A2b1	A2b2	A2b2111	A2b2180	B	B1	B2
Cameroon	CMR	Africa	15	11	0	0	0	0	11	0	0	4	1	3
Gambia	GMB	Africa	24	0	0	0	0	0	0	0	0	24	22	2
Kenya	KEN	Africa	99	52	0	0	0	41	11	0	0	47	29	18
Mali	MLI	Africa	30	0	0	0	0	0	0	0	0	30	0	30
Rwanda	RWA	Africa	1	0	0	0	0	0	0	0	0	1	0	1
South Africa	ZAF	Africa	80	41	29	0	10	1	1	0	0	39	16	23
Zambia	ZMB	Africa	11	1	0	0	1	0	0	0	0	10	9	1
China	CHN	Asia	131	91	5	0	1	5	46	27	7	40	23	17
India	IND	Asia	64	45	0	0	0	15	12	11	7	19	5	14
Iran	IRN	Asia	8	5	0	0	0	0	5	0	0	3	3	0
Iraq	IRQ	Asia	16	0	0	0	0	0	0	0	0	16	0	16
Japan	JPN	Asia	146	86	1	0	2	8	32	27	16	60	24	36
South Korea	KOR	Asia	1	0	0	0	0	0	0	0	0	1	1	0
Kuwait	KWT	Asia	1	0	0	0	0	0	0	0	0	1	0	1
Laos	LAO	Asia	6	5	0	0	0	0	5	0	0	1	1	0
Malaysia	MYS	Asia	69	30	0	0	0	15	15	0	0	39	24	15
Nepal	NPL	Asia	39	14	0	0	0	11	3	0	0	25	11	14
Saudi Arabia	SAU	Asia	9	4	0	0	1	3	0	0	0	5	3	2
Singapore	SGP	Asia	9	3	0	0	3	0	0	0	0	6	0	6
Thailand	THA	Asia	1	1	0	0	0	0	1	0	0	0	0	0
Germany	DEU	Europe	1	1	1	0	0	0	0	0	0	0	0	0
Spain	ESP	Europe	162	49	0	0	0	0	0	12	37	113	60	53
Finland	FIN	Europe	4	4	4	0	0	0	0	0	0	0	0	0
United King-dom	GBR	Europe	2	1	0	0	0	1	0	0	0	1	1	0
Greece	GRC	Europe	24	0	0	0	0	0	0	0	0	24	0	24
Croatia	HRV	Europe	67	46	0	0	2	10	15	6	13	21	9	12
Netherlands	NLD	Europe	32	17	3	3	4	6	1	0	0	15	7	8
Norway	NOR	Europe	2	2	0	0	1	0	1	0	0	0	0	0
Canada	CAN	North America	145	87	32	0	22	30	3	0	0	58	8	50
Mexico	MEX	North America	3	1	0	0	0	1	0	0	0	2	2	0
Nicaragua	NIC	North America	1	0	0	0	0	0	0	0	0	1	0	1
United States of America	USA	North America	107	64	15	11	7	11	20	0	0	43	12	31
Australia	AUS	Oceania	49	27	12	0	3	8	2	1	1	22	11	11

Argentina	ARG	South America	23	9	5	0	4	0	0	0	0	14	7	7
Brazil	BRA	South America	11	10	1	0	0	0	0	0	9	1	0	1
Peru	PER	South America	64	53	0	0	10	35	8	0	0	11	5	6
Uruguay	URY	South America	5	4	1	0	3	0	0	0	0	1	0	1
